# Integrating Welfare Technology in Long-term Care Services: Nationwide Cross-sectional Survey Study

**DOI:** 10.2196/22316

**Published:** 2021-08-16

**Authors:** Hanne Marie Rostad, Randi Stokke

**Affiliations:** 1 Department of Health Sciences in Gjøvik Faculty of Medicine and Health Sciences Norwegian University of Science and Technology Gjøvik Norway

**Keywords:** ambient assisted living, cross-sectional survey, home care services, innovation, long-term care, nursing homes, telecare, welfare technology, mobile phone

## Abstract

**Background:**

Welfare technologies are often described as a solution to the increasing pressure on primary health care services. However, despite initiating welfare technology projects in the health care sector and different government incentives, research indicates that it is difficult to integrate welfare technology innovations in a complex and varying setting, such as long-term care.

**Objective:**

We aim to describe the types of welfare technology and the extent to which welfare technology is provided in long-term care (ie, nursing homes and home care services); examine whether the extent of welfare technology provision differs on the basis of municipal characteristics (ie, population size, centrality, the proportion of older inhabitants, and income); and identify how local governments (ie, municipalities) describe their efforts toward integrating welfare technologies in long-term care.

**Methods:**

Quantitative and qualitative data about welfare technology from a larger cross-sectional survey about the provision of long-term care services in Norwegian municipalities were combined with registry data. Representatives of 422 Norwegian municipalities were invited to participate in the survey. Frequencies were used to describe the distribution of the types and extent of welfare technologies, whereas the Fisher exact test and Kruskal-Wallis one-way analysis of variance were used to determine the association between the extent of welfare technology and municipal characteristics. Free-form text data were analyzed using thematic analysis.

**Results:**

A total of 277 municipalities were surveyed. Technology for safety was the most widespread type of welfare technology, whereas technology for social contact was the least prevalent. Two-thirds of the sample (183/277, 66.1%) in nursing home and (197/277, 71.1%) in home care services reported providing one or two different types of welfare technology. There was a statistically significant association between the extent of welfare technology and population size (in both nursing homes and home care services: *P*=.01), centrality (nursing homes: *P*=.01; home care services: *P*<.001), and municipal income (nursing homes: *P*=.02; home care services: *P*<.001). The extent of welfare technology was not associated with the proportion of older adults. The municipalities described being in a piloting phase and committing to future investment in welfare technology. Monetary resources were allocated, competency development among staff was initiated, and the municipalities were concerned about establishing collaborations within and between municipalities. Home care services seem to have a more person-centered approach in their efforts toward integrating welfare technologies, whereas nursing homes seem to have a more technology-centered approach.

**Conclusions:**

Many municipalities provide welfare technologies; however, their extent is limited and varies according to municipal characteristics. Municipal practices still seem dominated by piloting, and welfare technologies are not fully integrated into long-term care services. Innovation with welfare technology appears top-down and is influenced by national policy but also reflects creating a *window of opportunity* through the organization of municipal efforts toward integrating welfare technology through, for example, collaborations and committing personnel and financial resources.

## Introduction

### Background

Owing to demographic changes and prolonged living expectations, most Western societies continue to face an increasing population of older adults [1,2]. This has contributed to a policy focus on active and independent living for older and frail adults [3,4]. In addition, the decentralization of health care services and the financial pressure to limit public costs have led to increasing pressure to develop sustainable primary health care services [5-7] because the status quo cannot be maintained [8].

As health care systems and terminology vary across countries, we must clarify the terms *primary health care* and *long-term care*. We define *primary health care* as a broad term that covers health care services throughout an individual’s life span, ranging from prevention to treatment, rehabilitation, and palliative care [9]. In Nordic countries, primary health care is predominantly publicly funded at the municipal level [10], the atomic unit of local government in Norway; municipalities vary significantly in size, topography, and demography, resulting in different priorities in the provision of primary health care services.

*Long-term care* is provided in municipal primary health care and involves services specifically directed at people who need assistance to perform basic activities of daily living. Various services are provided by different caregivers to address both medical and nonmedical needs at home, in assisted living facilities, or in nursing homes [11]. In Norway, municipalities are legally responsible for financing and providing long-term care services, such as home care and nursing homes—services found in every municipality—that are primarily funded through so-called *unrestricted revenues per capita*, consisting of tax revenues and block grants from the central government.

For governments in Western countries, innovation offers a potential *solution* to the aging population, the diminishing workforce, and increased demand for primary health care services [12]. Innovation is often described as a new product or service that represents a significant change for the people involved [13], which is integrated into practice and can be repeated and translated into new contexts [14,15]. Different technological devices are important examples of innovation and are both products and integrated parts of the service through innovation processes. Welfare technologies interact with the people involved in the service; they not only support care, but also change how care is provided and different people’s roles and responsibilities [16].

Although many terms can be used to reference technological innovations, in this paper, we refer to them as *welfare technology*. *Welfare technology* is an umbrella term, mainly used in Nordic countries, that covers technologies that have the potential to maintain or improve individuals’ functioning, safety, and independence, thereby promoting their well-being and reducing the need for formal and informal care. Other commonly used terms are *telecare*, *telehealth*, *ambient assisted living technologies*, *telemedicine*, and *eHealth*. Although these terms cover different forms of digital care, there is a considerable overlap among them [17,18].

A commitment to welfare technology is prominent throughout the European Union as underpinned by the European Commission’s communication on “Telemedicine for the Benefit of Patients, Healthcare Systems and Society” [8]. Western societies and their governments have allocated generous funding to promote technological innovations in care services [17]. This commitment is demonstrated by a series of official government documents in Norway [19-21]. In 2014, the government launched a program for welfare technology innovations in long-term care services—the National Welfare Technology Program—aimed at increasing the focus, investment, and integration of welfare technology in long-term care services [22]. In this paper, the term *integration* is defined as the process of welfare technology becoming a part of the municipality’s end-to-end long-term care services to meet the municipality’s goals and requirements for long-term care provision. The program is described in detail in Multimedia Appendix 1 [23-25].

Politicians view public innovations as a necessity for prosperity and progress in society [22], and technological innovations in long-term care are high on the agenda of Western societies [26,27]. The Norwegian government aimed to integrate welfare technologies into long-term care services by the end of 2020, and many municipalities have received funding from the government over the last decade to pilot various types of welfare technologies in their care services [28,29]. The current status of welfare technology integration in long-term care services is not known; however, previous research has shown that despite promising results, many welfare technology projects do not pass the pilot stage [30-33]. Consequently, several studies on technology in caring practices have involved pilot studies assessing the drivers of and barriers to integration of such technology into regular use [34,35]. However, these studies do not provide insights into how local governments perceive and organize their efforts toward integrating welfare technology in care services and whether integration varies according to the characteristics of local governments. This study addresses these research gaps using new data from Norwegian municipal long-term care settings. We intend to contribute to the empirical knowledge of welfare technology innovation practices in local governments and provide theoretical insights into how such innovation processes occur in long-term care services.

### Theoretical Framing

Studies of technologies emanates from different interdisciplinary academic disciplines [36]. We draw inspiration from Nicolini [37] and on what he describes as a *theory-method package*. This involves zooming in on the practice of integrating welfare technologies in municipal long-term care services and zooming out following trails of connections. Nicolini [37] argues that the purpose of social science is to open a rich and nuanced understanding of the practice and that there is no such thing as a unified practice theory. In this paper, we use what he calls a *toolkit approach* by mobilizing the aspects of different theories when exploring practice, such as theory of public service innovation and those from science and technology studies. Innovation studies and science and technology studies complement each other by focusing on the analysis of the role of technology and the use of technologies in innovation processes [38]. Sharing common origins, both fields have a high degree of interdisciplinarity and focus on how processes unfold, how society is structured, and how innovations evolve in society [39]. A major difference between innovation studies and science and technology studies is that innovation researchers tend to focus and development and look for solutions to problems in management or policy, whereas science and technology studies have a more critical approach, focusing more on the consequences of technology innovation processes [22,40]. Combining these fields enables us to provide an enriched understanding of what is happening. It also provides perspectives and ideas to further explore and understand welfare technology integration in long-term care services in local governments.

Innovations with technology aim to increase the effectiveness and efficiency of the public sector. However, innovation via technology and innovation [41,42] in a long-term care setting and in care work in general [27,43,44] are highly complex, involving *wicked problems* [45,46], such as many and changing stakeholders, competing interests, and disagreements regarding the nature of the problem [47,48]. Star and Ruhleder [16] introduced the importance of infrastructure for technology integration, which was later revisited by Greenhalgh et al [49]. Common metaphors describe infrastructure as “something upon which something else ‘runs’ or ‘operates’” (eg, physical structures, such as railroad tracks upon which rail cars run) [16]. However, Star and Ruhleder [16] and Greenhalgh et al [49] have argued that infrastructure also includes organizational (eg, rules, routines, processes, practices, and norms) and relational (eg, the relationships between the actors involved, as well as the practices and technologies) features, which generate particular agendas and priorities that influence technological innovation and its integration.

### Aim and Objectives

This study aims to provide knowledge on the current status of welfare technology integration in long-term care services and how local governments perceive and organize their efforts toward integrating welfare technology.

The study objectives are three-fold: (1) to describe the types and extent to which welfare technology is provided in long-term care; (2) to examine whether the extent of welfare technology provision differs on the basis of municipal characteristics (ie, population size, centrality, the proportion of older inhabitants, and income); and (3) to identify how local governments (ie, municipalities) describe their efforts toward integrating welfare technologies in long-term care.

## Methods

In this study, we applied data triangulation, which is the “use of different sources of data as distinct from using different methods in the production of data” [50]. Quantitative and qualitative data from a cross-sectional survey of long-term care settings (ie, nursing homes and home care services) in Norway were combined with registry data from publicly available national statistics.

### Setting and Participants

When we conducted our study in 2019, Norway had 422 municipalities distributed among five regions; however, this number declined to 356 municipalities in 2020 due to regional and municipal reforms [51]. Total population sampling was used, a type of purposive sampling technique [52], in which all Norwegian municipalities were invited to participate. The municipalities were contacted via email and asked to choose one person with extensive knowledge of the municipality’s long-term care services to answer the survey on behalf of their municipality. The title of the selected person varied due to the varying organizational structures among municipalities. The varying titles meant varying functions, tasks, responsibilities, and the degree to which the respondent could make decisions in their organization: some made long-term decisions on a strategic level, whereas others decided day-to-day operations on an operational level. The person designated as the respondent was contacted via email, which provided information about the study and participation, in addition to a link to the survey. To encourage participation, the email described how the study could contribute to improved knowledge and understanding of current practices, which could aid both local and national policy makers who plan, set priorities, and develop services for the future, ultimately improving long-term care services for patients and their families. Email reminders were sent three times, approximately every 4 weeks, during the study period.

### Data Sources

A web-based questionnaire was designed based on a comprehensive review of the literature. We developed the questionnaire together with a user panel consisting of representatives from five municipalities of different sizes (based on population) and geographical locations. The representatives held different positions as leaders and advisers at different organizational levels but all worked in or for long-term care services. In addition, the representatives in the panel helped establish face validity and reviewed whether the questionnaire effectively captured the topic under investigation and checked for double, confusing, or misleading questions. None of the representatives in the panel participated in the study on behalf of their municipality. In addition to the user panel, the survey was piloted by 3 representatives from the target group, 2 of which also answered the main survey, and their responses were included in the analyses. Adjustments were made to the survey based on the feedback from the user panel and pilot. Specifically, questions were rephrased or removed due to a lack of relevance.

A more detailed description of the questionnaire is provided in Multimedia Appendix 2. As we used conditional branching in the survey, the respondents may take different paths through the survey depending on their answers; not everyone received all the questions. The participants were able to start, stop, and resume answering the questionnaire until the survey was closed. Two close-ended questions were analyzed in this study: what types of welfare technology were provided in (1) nursing homes and (2) home care services? Two open-ended questions allowed the respondents to elaborate on integrating welfare technology in nursing homes and home care services. Data were collected between February and April 2019.

### Variables

Questions about the types of welfare technology in nursing homes and home care services were close-ended questions with a predefined list of answers, wherein the respondents could check off all the choices that applied to them. The predefined list was based on the categorization in the official government document “Innovation in the Care Services” [53] (Textbox 1).

**Categories of welfare technologies.**

**Categories and examples**

- Localization technologies
  GPS
- Compensation technologies
  Remote control of light and heating, robot vacuums, and cognitive or physical aids
- Safety technologies
  Social alarm and fall detection sensors
- Technologies for social contact
  Tablets, smartphones, gaming, and therapeutic robots
- Treatment technologies
  Medical remote monitoring and automated pill dispensers

The extent of welfare technology was measured as the number (ranging from 0 to 5) of the different types of welfare technologies provided by the municipality. Data on municipal characteristics included population size, centrality, the proportion of older adults, and municipal income, which were obtained from publicly available statistics (Statistics Norway):

- Population size has three categories, as follows: small (<4999 inhabitants), medium (5000-19,999 inhabitants), and large (>20,000 inhabitants) [54]. Data were from the first quarter of 2019.
- Centrality has three categories, as follows: least central, central, and most central. Data were from January 2018 and based on Statistics Norway’s centrality index. The centrality index was based on the travel time to workplaces and service functions (eg, post office and bank) [52].
- The proportion of older adults was a continuous variable for the percentage of the municipality’s inhabitants aged ≥80 years in 2019.
- Municipal income was measured as “unrestricted revenues per capita,” which is a continuous variable for how much income the municipalities have at their disposal after covering the fixed costs, indicating the municipalities’ financial leeway. Data were from the first quarter of 2019.

### Data Analysis

Quantitative data were analyzed using SPSS version 25 (IBM Corporation). Frequencies were used to describe the distribution of the types and the extent of welfare technologies provided in nursing homes and home care services. The differences between the responders and nonresponders in terms of municipal characteristics were tested using a chi-square analysis and two-tailed *t* test for independent samples. The Fisher exact test was used to determine the association between the extent of welfare technology and population size and centrality, whereas the Kruskal-Wallis one-way analysis of variance was used to determine the association between the extent of welfare technology and the proportion of older adults and municipal income. The free-form text data from the open-ended survey questions were analyzed using the thematic analysis by Braun and Clarke [53], as described in Multimedia Appendix 3 [53]. Qualitative data were analyzed manually, resulting in the following four themes: (1) from good intentions to established reality, (2) investments in and rigging up the welfare technology initiative, (3) type of technology they are going for, and (4) rationale for focus and selection.

### Ethical Considerations

Before the initiation of data collection, the Data Protection Authority within the Norwegian Centre for Research Data assessed the study procedure and concluded that the processing of personal data in this study was in accordance with privacy legislation (reference no. 847216).

An informed consent form was attached to the email sent to the potential respondents, stating that the person consented to participate in the study by completing the survey. Participation was confidential. The participating municipality (not the individual completing the questionnaire) was identified only by the researchers.

## Results

### Overview

A total of 65.6% (277/422) of municipalities completed the survey. There was a large geographical spread among the responding municipalities, and all five regions were represented (Table 1).

Responders and nonresponders were compared in terms of population size, centrality, proportion of older adults, and municipal income. The smallest municipalities (in terms of population size) were underrepresented (Table 2). The responders had a lower proportion of older adults and lower mean municipal income than nonresponders. In terms of the open-ended questions, there were 170 responses out of 554 possible responses (277 municipalities×2 questions).

**Table 1:** Geographical spread of responders.

Region	Municipalities in the regions, n (%)	Municipalities participating in the study, n (%)
Northern	87 (20.6)	44 (15.9)
Mid	48 (11.4)	33 (11.9)
Western	120 (28.4)	81 (29.2)
Southern	30 (7.1)	18 (6.5)
Eastern	137 (32.5)	101 (36.5)

**Table 2:** Comparison of responders and nonresponders (N=422).

Variable		Responders (n=277)	Nonresponders (n=145)	*P* value	
**Population size,** **n (%)**					.03
	Small	131 (47.3)	88 (60.7)		
	Medium	105 (37.9)	39 (26.9)		
	Large	41 (14.8)	18 (12.4)		
**Centrality,** **n (%)**					.20
	Most central	23 (8.3)	7 (4.8)		
	Central	106 (38.3)	49 (33.8)		
	Least central	148 (53.4)	89 (61.4)		
Older inhabitants (%), mean (SD)		5.2 (1.4)	5.6 (1.5)	.01	
Municipal income (NOK^a^), mean (SD)		62,567.9 (12,705.6)^b^	66,730.0 (13,956.1)^c^	.01	

^a^NOK: Norwegian Kroner.

^b^Equivalent to a mean of US $7669.1 and an SD of US $1557.4.

^c^Equivalent to a mean of US $8179.4 and an SD of US $1710.7.

### Types and Extent of Welfare Technology

Independent of setting, technology for safety was the most widespread welfare technology provided, followed by localization technology. Almost all respondents, 96% (266/277), reported having technologies for safety in home care services, whereas 81.9% (227/277) reported them for nursing homes. Examples of technologies for safety include social alarms, fall detectors, bed and chair sensors, and digital supervision. Technology for social contact was the least prevalent, provided by 11.6% (32/277) in nursing homes and 9.7% (27/277) in home care services. Examples of social technology include videoconferences and therapeutic robots.

The thematic analysis of the participants’ open-text responses revealed that in the home care setting, the municipalities chose both *types* of technology and selected certain groups to receive the technology:

The municipality offers follow-up through telemedicine for patients with chronic obstructive pulmonary disease and diabetes.

Respondent from a small, least central municipality with an average proportion of older adults aged ≥80 years and a high income

This quote illustrates how the municipalities directed their focus to particular user groups, such as people with different kinds of chronic diseases.

With regard to the extent of welfare technology, most respondents reported having only one or two different types of welfare technology, irrespective of the setting (Table 3).

**Table 3:** Types and extent of welfare technologies available.

Variable		Nursing home	Home care services
**Types, n (%)**			
	Localization	137 (49.5)	129 (46.6)
	Compensation	48 (17.3)	50 (18.1)
	Safety	227 (82)	266 (96)
	Social contact	32 (11.6)	27 (9.8)
	Treatment	35 (12.6)	73 (26.4)
**Extent, n (%)**			
	None	36 (13)	8 (2.9)
	1	88 (31.8)	102 (36.8)
	2	95 (34.3)	95 (34.3)
	3	40 (14.4)	44 (15.8)
	4	13 (4.7)	21 (7.6)
	5	5 (1.8)	7 (2.5)

### Variations According to Municipal Characteristics

The extent of welfare technology varies according to municipal characteristics, including size, centrality, and income. Surprisingly, our data did not show an association with the proportion of older adults. These variations can be both natural and justifiable, but also contribute to some challenges—something we will come back to in the discussion chapter.

There was a statistically significant association between the extent of welfare technology, population size, and centrality in both nursing homes and home care services. A larger percentage of the largest, most central municipalities seem to provide more types of welfare technologies than the smallest, least central municipalities (Tables 4 and 5).

**Table 4:** Association between the extent of welfare technology in nursing homes and population size and centrality.

Variable		None	1	2	3	4	5	*P* value	
**Population size, n (%)**									.01
	Small	21 (16)	45 (34.4)	45 (34.4)	17 (12.9)	3 (2.3)	0 (0)		
	Medium	12 (11.4)	37 (35.2)	37 (35.2)	11 (10.5)	5 (4.8)	3 (2.9)		
	Large	3 (7.3)	6 (14.6)	13 (31.7)	12 (29.3)	5 (12.2)	2 (4.9)		
**Centrality, n (%)**									.01
	Most central	3 (13)	2 (8.7)	7 (30.4)	8 (34.8)	3 (13)	0 (0)		
	Central	11 (10.4)	34 (32.1)	37 (34.9)	13 (12.3)	6 (5.7)	5 (4.7)		
	Least central	22 (14.9)	52 (35.1)	51 (34.5)	19 (12.8)	4 (2.7)	0 (0)		

**Table 5:** Association between the extent of welfare technology in home care services and population size and centrality.

Variable			None		1		2		3		4		5		*P* value	
**Population size, n (%)**																.01
	Small	5 (3.8)		63 (48.1)		4 (3.1)		17 (13)		4 (3.1)		2 (1.5)				
	Medium	2 (1.9)		32 (30.5)		41 (39.1)		16 (15.2)		10 (9.5)		4 (3.8)				
	Large	0 (0)		8 (19.5)		14 (34.2)		11 (26.8)		7 (17.1)		1 (2.4)				
**Centrality, n (%)**																<.001
	Most central	1 (4.4)		5 (21.7)		4 (17.4)		7 (30.4)		6 (26.1)		0 (0)				
	Central	0 (0)		33 (31.1)		43 (40.6)		17 (16)		8 (7.6)		5 (4.7)				
	Least central	6 (4.1)		65 (43.9)		48 (32.4)		20 (13.5)		7 (4.7)		2 (1.4)				

There was a statistically significant association between the extent of welfare technology and municipal income in both nursing homes (*P*=.02) and home care settings (*P*<.001). Municipalities with a lower income provided more types of welfare technologies than those with a higher income. No statistically significant association was found between the extent of welfare technology and the proportion of older inhabitants in either nursing homes (*P*=.33) or home care settings (*P*=.50).

### From Good Intentions to Established Reality

This theme illustrates how far the municipalities have come in the process of integrating welfare technologies into their long-term care services, which varied considerably. A few municipalities described that welfare technologies were integrated into the municipality’s long-term care services, whereas status in most municipalities seemed well reflected in the following quote:

We have not integrated these types of solutions, but we take part in the National Welfare Technology Program and will pilot different types of welfare technology in 2019–2020.

Respondent from a small, least central municipality with an average proportion of older adults and a high income

Some municipalities described projects focused on updating established technologies, whereas most municipalities participated in projects concerning the integration of new technologies. Few respondents reported that welfare technology was integrated into their care services.

### Investing in and Rigging up the Welfare Technology Initiative

This theme concerns how municipalities, as local governments, perceived and organized the integration of welfare technology in their long-term care services. The municipalities appeared enthusiastic about integrating welfare technology as respondents described how they are committing themselves to further initiatives toward integrating more technology, both in terms of extending them to new patient groups and use in other services:

Automated pill dispensers will be offered to suitable users in 2019, as well as increased diffusion of safety and sensor technology to more users, for example, for localization, falls, etc...The municipality is now tendering for welfare technology solutions—initially for purchasing new social alarms. More welfare technological solutions will eventually come.

Respondent from a small, least central municipality with an above average proportion of older adults and a high income

Other municipalities described how they are preparing for future investments in welfare technology:

We have allocated funds in the financial planning period to invest in welfare technology; NOK 500,000 [approximately US $60,000] in 2019, and NOK 1 million [approximately US $120,000] for each of the three upcoming years.

Respondent from a medium, least central municipality with an average proportion of older adults and a high income

Some municipalities described how they invest in competency development among staff and that they have staff that are dedicated to working with welfare technology investment regarding mapping needs and what is available:

The municipality is at the starting point with welfare technology, with continuing education and basic training of staff.... We have employed a dedicated resource person/adviser in welfare technology.

Respondent from a large, central municipality with an average proportion of older adults and a low income

In terms of timing, the investment was done when certain opportunities presented themselves, often involving various demands concerning the necessary attributes of welfare technologies:

The municipality is in a phase of constructing new assisted living facilities and a new nursing home, general practitioners’ office, physio/occupational therapy, health, and mental health center. A lot of time and resources are used to create good welfare technology solutions for the future. Two people from IT are included in the project group.

Respondent from a small, least central municipality with an above average proportion of older adults aged ≥80 years and a low income

The acquired welfare technology must be cloud-based, provident, and scalable, and all notifications are received on the same response unit. New sensors/technology will be connected in line with patient needs.

Respondent from a small, least central municipality with an above average proportion of older adults aged ≥80 years and a low income

As illustrated above, the municipalities were also concerned with intersectional collaboration, for example, between the municipality’s care and information technology sectors. In addition, many municipalities described an intermunicipal collaboration concerning the initiation and execution of welfare technology projects, and some collaborated on assessing the needs for welfare technology investment, inviting tenders, and investing in welfare technology for common use.

### Rationale for Investing in Welfare Technology Solutions

The rationale for investing in welfare technologies in nursing homes was different from that for home care services. In home care, the choice of technology depended on an individual patient’s needs, whereas in nursing homes, the choice was directed at a collective service *offered* to every resident. This indicates a difference in perspectives and approaches—user-centered versus technology-centered. Home care services were also concerned with who was responsible for what types of technology:

We are concerned with the individual’s freedom and responsibility to be able to buy technology they feel they need for themselves. We believe that it is not a matter of course that all welfare technology should be offered by the municipality. Safety technology and technology that can replace supervision (digital supervision)—yes, but automated lighting and heating? No, I mean private builders and private homeowners must be able to take responsibility for themselves.

Respondent from a medium, central municipality with a below average proportion of older adults and a high income

In contrast, in nursing homes, technology was linked to the institution, with a focus on general use:

The building will be equipped with alert technology, video communication, digital surveillance, and remote monitoring.

Respondent from a medium, central municipality with an average proportion of older adults and a medium income

## Discussion

### Principal Findings

By describing the types and extent to which welfare technology is provided in nursing homes and home care services and determining whether the extent of welfare technology provision differs according to municipal characteristics (the *what* and the *who*), this study aimed to provide knowledge of the current status of welfare technology integration in long-term care services and how local governments perceive and organize their effort toward integrating welfare technology. Furthermore, this study identified how local governments (ie, municipalities) describe their efforts toward integrating welfare technologies in long-term care (the *how* and the *why*).

### The What and Who of Technology Innovations in Long-term Care Services

Our findings suggest that welfare technologies are widespread in Norwegian home care services and nursing homes as most municipalities provided them. However, the extent was not great; most municipalities provided only one or two different types of welfare technologies.

The reported welfare technologies for safety provided by the broad number of municipalities most likely refers to social alarms, which are standard in Norway [55] and widely used in other Western societies [56]. Norwegian health authorities recommend municipalities to invest in technologies for safety, along with localization technology, because “the reward is evident” [55]. Accordingly, our findings showed how a national policy initiates local innovation and how a national policy becomes a local priority. Considerable amounts of innovation in public services and frontline work processes have been initiated by the central levels of government with the intention that they are implemented in a top-down manner and disseminated at the frontline level of public service organizations [57]. In this way, national governments can initiate innovation processes through policy documents, regulations, and funding, and the local government, which is closer to citizens, can initiate, develop, and activate the innovation processes [58]. Thus, local governments are both tools for state governance and an arena for local governance [59]. The Norwegian Welfare Technology Program is an example of how the central government uses *soft measures*, such as financial initiatives, to allow local governments to decide whether they will respond to financial stimulants and political signals. Previous research on government reform efforts to establish primary health care services (ie, local medical centers) has indicated that government funding has great significance for local investment and that municipal investments are more influenced by the possibility of state funding than a real need for services [60]. Thus, the question of how central government funding schemes impact real local self-government is relevant for further research.

Our finding that safety technology is the most prevalent type can also be explained by the fact that ensuring patient safety is a top priority in global health [54] and a key expectation for welfare technology from the perspectives of both the government [19] and care professionals [61]. Furthermore, the concept behind welfare technology is remote patient monitoring, focusing on the personal safety of the individual [62]. Although providing welfare technologies is not required by the law, the Norwegian government strongly encourages municipalities to integrate them into their health care services. The services required by law are the municipalities’ main priority, making innovation and service development through welfare technology somewhat secondary. Thus, municipalities would acquire certain types of technologies with clear potential for more efficient services and cost savings, such as safety technologies, rather than social technologies. For instance, digital supervision, such as through fall sensors, can reduce the need for regular checkups in the patient’s room or home and thus the number of staff per shift, whereas a video communication device might not have the same efficiency potential. Furthermore, a study on Norwegian nursing homes found that registered nurses rarely had any time to address the residents’ psychosocial needs because they felt that they had to prioritize their medical and physiological needs [63]. Social needs, such as social contact and belongingness, are basic human needs and are a particular focus of nursing practice and a key task for long-term care services. However, social needs may largely be taken care of by long-term care recipients themselves through devices that are widespread in today’s society, such as smartphones and tablets. As such, the technology for social contact may have already been acquired by the individual care recipient, which may also explain why only a few municipalities provided this category of technology in long-term care.

Following this notion, our findings bring forward an interesting question of *responsibility*, indicating the ongoing debate about the extent to which local governments should be responsible for providing technologies for their inhabitants and what the individuals themselves should acquire. In Nordic countries with large public sectors, there has been little tradition of individual investments in welfare technologies. However, having the current and future users of primary health care services take more responsibility for their own health and well-being is a key goal of an ongoing social reform in Norway called “A Full Life—All Your Life—A Quality Reform for Older Persons” [64], one aim of which is to make the user more accountable by, for example, customizing and equipping their own homes to facilitate independence.

Another finding is the statistically significant association between the extent of welfare technology and population size and centrality in both nursing homes and home care services. The largest and most central municipalities provided more types of different technologies than the smallest and least central municipalities. One possible explanation may be that larger, more central municipalities have a larger number and a more continuous flow of patients with complex chronic diseases, which demand more resources. Thus, providing certain services, such as welfare technology, is likely to be more sustainable in larger, more central municipalities than in those with fewer patients. In addition, larger municipalities have different competence compositions and other resources [65], possibly impacting their capacity to apply for government project funding. Urban areas also have some distinguishing features that are important for innovation capacity: density and diversity [66], where “...density creates a constant flow of new information that comes from observing others successes and failures” [67] and where population diversity means that there are different challenges, needs, resources, experiences, cultural and religious backgrounds, creating an environment wherein *out-of-the-box* ideas are more likely to occur [66]. On the other hand, the benefits of welfare technology may be better appreciated by caregivers operating in rural areas because this technology can remedy large travel distances [68] and provide rural residents better access to chronic disease prevention and quality of care [69,70].

However, the potential benefits of different types of welfare technology differ according to municipal characteristics, such as centrality, population size, and demographics, and the population’s health and care needs. For example, the technology for optimizing route planning in home care services is likely more beneficial in large, central municipalities with more traffic and several route options than in small rural municipalities. On the other hand, medical remote monitoring may have more benefits in small rural municipalities. When integrating welfare technologies into different contexts, we expect the same processes and effects. This is problematic because we simplify the complexity and wicked problems. The examples above illustrate how we must always consider the context because welfare technology integration always involves interactions between technology and humans within a context [34].

We also found that municipalities with lower income provided more types of welfare technologies than those with higher incomes. This is likely explained by the fact that innovation in services as an increased use of welfare technology is driven by the strained economy of local governments [71]. Local governments with large financial leeways may not need new or different ways of producing long-term care services because they can afford to maintain the status quo, whereas those with a more constrained financial situation need new solutions to sustain the required standards of care. As such, necessity is the mother of invention.

There is a possible additional aspect concerning the association between the extent of welfare technology and municipal income: can welfare technology be viewed as a form of *second-class* care? In debates regarding welfare technology, some researchers have asked whether *cold* technologies will be integrated at the cost of *warm* human care [72]. Welfare technologies affect the service provided and might be seen as an antagonist to traditional *warm* care. Furthermore, questions have been raised about whether the use of welfare technology creates a stigma because it may signal an inability to master everyday things [73], hence looking and feeling old or vulnerable [74,75], even though the aim is independence. Thus, there may be some reluctance from local stakeholders, such as politicians and leaders of long-term care services, to introduce value-changing care innovations if they are not *forced* to do so by, for example, a strained municipal economy.

The fact that a higher proportion of adults aged above ≥80 years was not associated with the provided welfare technology was somewhat surprising, as welfare technology is proposed as a potential *solution* to the age wave [12] and that much of the welfare technology developed is for this user group. However, this may indicate that *age* is not important for the municipalities’ provision of welfare technology. Municipalities that invested in technology aimed at specific user groups, such as patients with dementia and chronic obstructive pulmonary disease, did likely do so due to the individual’s physical and cognitive function, rather than the individual’s age. Reduced physical and cognitive function results in reduced independence, entailing high costs for the individual, family, and society, whereas health care spending on healthy and independent older adults is relatively modest [76]. Caring for people affected by chronic obstructive pulmonary disease, stroke, and dementia—prevalent patient groups in long-term care—are resource-draining and welfare technologies with potential for more efficient services and cost savings are therefore worth investing in.

As discussed, the extent of welfare technology seems to vary according to municipal characteristics, which is justifiable for several reasons. As the municipalities significantly varied in terms of size, topography, and population composition, different needs must be met when providing care to their inhabitants. Thus, the potential of welfare technology in providing care will depend on many different factors, a limited number of which were explored in this study. However, an important question might be whether the differences in welfare technology provision can threaten the founding principles of many health care systems, such as universality and horizontal equity (ie, equal treatment of individuals or groups in the same circumstances), leading to variations in the quality of care.

### The How and Why—the Window of Opportunity

Our results indicate that the municipalities were far from achieving the government’s goal of integrating welfare technology into Norwegian long-term care by the end of 2020. Most municipalities described how they were involved in projects or pilot testing. Over the past two decades, there has been a steady growth in the number of technological innovation projects in the health care sector [30]. Both policy makers and researchers have raised concerns over pilots in eHealth and telemedicine, calling for the large-scale integration of technology in routine health service delivery [30]. A *plague of pilots* has been conducted [77], in which projects were established to be run as nonpermanent tests rather than integrated into routine service [30].

One reason for this is that it is always difficult to integrate new technologies within existing organizations, as they complicate complex daily care practices. The integration of new technology implies a change in the interactions and relationships in the organization that brings in new ideas, actors, tasks, and organizational changes in the service. Care work already largely involves actors in the network needing to adjust their practices or technologies [43,74]. In addition, although the municipalities receive incentives from the national government, they have many reasons to be careful when integrating new welfare technologies into their services. There is always a risk of failure that results in losing money with innovations, and municipalities are governed by the rules of democracy and laws [46]. Innovation might also not lead to improvement [14], and although they could lead to improvement, we must always ask “for whom is this an improvement?” In addition, public service has multiple aims that are sometimes conflicting, aiming to provide public and individual values [75].

Although welfare technology does not currently seem integrated into Norwegian long-term services, we interpret the municipalities as being in a *window of opportunity*, which represents a situation in which an established regime becomes unstable (because of external factors or internal problems) and is receptive to alternative regimes and innovation [78]. One example of how municipalities may be viewed as being in a window of opportunity is the current societal and health care reforms and associated financial schemes that encourage municipalities to innovate [79,80], for example, within welfare technology. Other examples are how municipalities are rigging up their welfare technology initiatives: they are entering into intermunicipal collaborations. The collaborative approach in welfare technology initiatives is a major facilitator of possible innovations [81,82]. In addition, collaboration entails shared costs and responsibilities that may result in more efficient and sustainable service solutions, especially for small municipalities [83]. The mode of operation within and among municipalities becomes more comprehensive and differentiated, but also more complex because power may become more elusive as decisions are made in larger networks, decisions for each individual municipality are made through negotiations across several municipalities, and political accountability may be difficult to determine [83]. The health care system is widely recognized as a complex system [84] loaded with wicked problems; thus, innovation in this setting is inherently complicated. The complexity within the system will shape its ability to create a window of opportunity to actively pursue change, adapt, and integrate innovation [85].

Furthermore, this study found that the municipalities are building competence in welfare technology by training and dedicating staff to the welfare technology initiative, indicating an ambition beyond just piloting. This indicates that the municipalities acknowledge that success with technological innovations takes more than just investing in technological devices. Many municipalities are also building and upgrading nursing homes, and they describe these processes as opportunities to invest in welfare technology in long-term care.

Geels and Schot [86] illustrated that transitions and system changes emerge through the interactions among processes at different levels (see the illustration by Geels and Schot [86] in Multimedia Appendix 4 [87]). Considering their theorization, we interpreted our data similarly: how public and technological innovation is shaped by different processes at different societal levels, for example, how demographic changes put pressure on current long-term care practice, thus opening a window of opportunity for innovation, such as welfare technology. This affects the development of national policy and may initiate changes in current practices, such as how the national welfare technology program (as national policy) is taken up by smaller units of actors (ie, municipalities) and further evolved there. The municipalities establish internal momentum through their local learning processes, which are conveyed back to the central government and can lead to adjustments, breakthroughs, and new windows of opportunity. This perspective can contribute to an understanding of how changes and ideas at one level trigger ideas and changes at other levels. This describes how innovation with welfare technology and its integration to its full potential are affected by human agency, social structures, and multilevel interactions.

Another interesting finding adding to the complexity of welfare technology innovations in long-term care is that the integration of welfare technology is characterized by different rationales in nursing homes and home care services. In home care settings, the inclusion of welfare technology was described as dependent on the wishes of the patients, whereas in nursing homes, this person-centeredness focus did not appear, and the focus was more directed at a collective service that every resident was *offered* and at a need for universal design. This can be explained by the different conditions and considerations when integrating welfare technology in nursing homes compared with home care services. Norwegian nursing homes function as a medical and health care institution, and a person moves there for a limited time until they die; in Norway, the median living time in nursing homes is 1.3 years [88], with a steady substitution of patients. Thus, it seems more sustainable to bring in technology linked to the institution rather than the individual patient, as technology in the nursing home setting needs to address universal needs and devices that would *fit* most people living in nursing homes. Furthermore, those who live in nursing homes inevitably face a significant reduction in the range of options available to them by the nature of institutionalized living itself, and an emphasis on the physical care of nursing home residents and a task-oriented approach may result in privileging efficiency over resident choice [89]. Although there has been a fundamental shift in thinking about nursing homes over the past two to three decades—now viewed as person-centered homes offering long-term care services rather than institutions [90], entering a nursing home still entails receiving care in a professional domain compared with home care services, where professionals enter the individuals’ private domain to provide care.

In summary, our discussion aims to illustrate how the integration of welfare technology innovations is formed by different factors, processes, and practices at different government levels (ie, national and local) as described by Geels and Schot [86]. These factors, processes, and practices are part of the infrastructure introduced by Star and Ruhleder [16] and Greenhalgh et al [49]. In this study, we analyzed factors such as municipal size and centrality, population factors, and municipal income. Furthermore, we discussed other factors such as policy, national government initiatives, and institutional norms and values, which are also factors included in the infrastructure that may affect the integration of welfare technologies. Focusing on infrastructure provides knowledge of how different factors, processes, and practices are inevitable parts of innovation. This aligns with what we know from the service innovation literature that innovation rarely can be diffused but must be translated to fit the new infrastructure. Thus, the results of studies like ours, focusing on welfare technology integration, will depend on the infrastructure and apply to the particular setting that the study aims to explore.

### Limitations and Recommendations for Further Studies

The organization of long-term care differs across countries, thus limiting the generalizability of our findings. However, we believe that our study provides new knowledge and relevant questions when addressing technological innovations in the public care sector in general.

In this paper, we studied the efforts to integrate welfare technology from the perspective of municipal managerial employees based on their knowledge and perceptions of the provision of welfare technology in long-term care services. As the respondents were not engaged in hands-on work, their knowledge of welfare technology provision may be incomplete.

As illustrated by Geels and Schot [86], innovation initiatives also occur in a bottom-up fashion. Therefore, it would be interesting for future studies to elicit the point of view of those who integrate such technologies into the daily operations of long-term care institutions and their users. In addition, this study was neither designed to investigate causality nor study behaviors, opinions, themes, and motivation in-depth. Therefore, in addition to investigating other settings and perspectives, future studies applying other designs and methods will be valuable in explaining and trying to understand the integration of welfare technology into health care services.

Although our sample covered 65.6% (277/422) of the entire Norwegian population of municipalities, small municipalities seemed somewhat underrepresented, whereas those with lower proportion of older adults and lower mean income appeared slightly overrepresented. Furthermore, the data for the open-ended questions were limited (only one-third of what could be obtained), so our results should be used with caution.

The categorization of welfare technology in this paper and Norwegian official government documents entails a significant overlap among the different types of technologies (eg, an automated pill dispenser can be both a treatment technology and a safety technology). Furthermore, the categories defined follow administrative logic and policy issues. Logics represent “frames of reference that condition actors’ choices for sense-making, the vocabulary they use to motivate actions, and their sense of self and identity” [91]. A politician, a long-term care nurse, and a long-term care recipient will all have varying assumptions, values, beliefs, and rules that shape their logic. Thus, the categorization of welfare technology used in this paper may need to be challenged and problematized in future studies because this understanding may have difficulty reaching the core functioning of long-term care when based on a logic emanating from policy goals and administrative rationale.

Considering the ongoing COVID-19 pandemic, studies focusing on whether (and how) the pandemic has affected investments in and use of welfare technologies in different health care settings would be of great interest, for example, whether the use of welfare technologies was introduced (eg, medical remote monitoring and social technologies) or technologies were used in new ways to compensate for the reduced possibility of physical presence in the service recipients’ place of residence, both on the part of formal and informal caregivers.

### Conclusions

This study has provided knowledge on the current status of welfare technology integration in long-term care services and insights into how local governments perceive and organize their efforts toward integrating welfare technology in the long-term care setting, thus contributing ideas into how technology innovation processes play out.

Many municipalities provide welfare technologies, whereas most provide safety technologies. However, their extent is limited and varies according to municipal characteristics. Welfare technologies do not seem to be fully integrated into Norwegian long-term care services because many are still in their project and piloting phases. However, the municipalities are motivated to invest in the welfare technology initiative. Therefore, we suggest the term *window of opportunity* as a way of providing insights into the potential for a real integration of welfare technologies rather than solely focusing on *pilotism* as a concern. *Window of opportunity* may be more appropriate for both technology innovations and long-term care services that are complex and innovation processes that take time and opportunity. In addition, innovations are shaped at different societal levels, affecting the ability to create a window of opportunity and facilitate innovation processes.

## Appendix

Multimedia Appendix 1 The National Welfare Technology Program in Norway.

Multimedia Appendix 2 Questionnaire.

Multimedia Appendix 3 The phases of the thematical analysis.

Multimedia Appendix 4 Geels and Schot processual framework.
