# Supplementary material for: Integrating Welfare Technology in Long-term Care Services: Nationwide Cross-sectional Survey Study
Source: J Med Internet Res. 2021 Aug 16;23(8):e22316. doi: 10.2196/22316 (PMC8406104; doi:10.2196/22316)
Supplement: Multimedia Appendix 1 [file jmir_v23i8e22316_app1.doc]

## Multimedia Appendix 1 - The National Welfare Technology Program in Norway

In 2013, the Norwegian government launched the Welfare Technology program. This program gave the Norwegian local governments – the municipalities – the opportunity to apply for funding for piloting, implementation and diffusion of technological innovation within municipal long-term care services.

The program was introduced to extend the use of welfare technologies in the long-term care services with the aim that welfare technologies would be integrated in all Norwegian municipalities within the year 2020.

# According to the Norwegian Directorate of Health, approximately 340 municipalities are participating in the program as of 2019. The expectations are that integrating welfare technology in care practices will provide a better services for both people living at home as well as for people living in nursing homes. Whether the municipalities chose to initiate projects for homecare services, nursing homes or both where based on their needs.

Different types of welfare technologies are included in the program and the Directorate of Health provide recommendation based on experiences and results from municipalities piloting these various technologies. Technologies such as GPS tracking system, automated pill dispensers, electronic door locks, digital supervision and digital and mobile social alarms are recommended as “the reward is evident”.

This information and more can be found here:

Brevik, K. 2014. A National Welfare Technology Programme in Norway, why and how? <https://www.stjornarradid.is/media/velferdarraduneyti-media/media/veltek2014/Kristine----A-National-Welfare-Technology-Program-Norway1.pdf>

The Norwegian Association of Local and Regional Authorities (KS). 2018. <https://www.ks.no/fagomrader/helse-og-omsorg/velferdsteknologi3/nasjonalt-velferdsteknologiprogram/>

The Norwegian Directorate of Health. 2019. <https://www.helsedirektoratet.no/tema/velferdsteknologi/velferdsteknologi>.
