# Supplementary material for: Integrating Welfare Technology in Long-term Care Services: Nationwide Cross-sectional Survey Study
Source: J Med Internet Res. 2021 Aug 16;23(8):e22316. doi: 10.2196/22316 (PMC8406104; doi:10.2196/22316)
Supplement: Multimedia Appendix 2 [file jmir_v23i8e22316_app2.doc]

# Multimedia Appendix 2 - Questionnaire

The questionnaire consisted of 35 close-ended possible questions and 49 open-ended possible questions where the respondents could elaborate on their answers. Because we used conditional branching, the respondent’s path through the survey varied based on their answers.

How long time the participants used to complete the survey varied greatly. We no longer have access to the system log data in the survey platform but have noted that the response time ranged from 10 minutes to approximately 120 minutes.

| Theme |  | Example of question |
| --- | --- | --- |
| Allocation of services |  | Who allocates long-term care services in your municipality?   - A separate unit for allocating services - Allocation takes place from the services themselves - Other, please specify |
| Provision of: |  |  |
|  | Nursing homes services | Some municipalities have specialized nursing home services for different patient groups. Please check the box if your municipality provides nursing homes placement for the following (choosing multiple options is possible):   - Dementia care - Reinforced dementia care - Neurological disorders - Psychiatric disorders - Substance abuse - None of these |
|  | Homecare services | * |
|  | Sheltered housing | * |
|  | Welfare technology | Check the box if your municipality provides one or more of the following types of telecare in nursing homes  (choosing multiple options is possible):   - Location technology/GPS - Compensation and wellness technology - Safety and security technology - Technology for social contact - Technology for (remote) treatment and care |
|  | Preventive and health-promoting services | Please check the box if your municipality provides one or more of the following offers for preventive and health-promoting services  (choosing multiple options is possible):   - Home visits for older adults who have limited/do not have care services - Organized physical activity - Individual conversations concerning living habits and health - Learning and mastery services for patients - Learning and mastery services for family members |
|  | Services for coordination of care | Please check the box if your municipality has a coordinator function for (choosing multiple options is possible):   - Dementia care - Oncological care - Palliative treatment/palliation - Coordination of patient care trajectory (e.g., between primary and secondary health service) - Habilitation/rehabilitation - Voluntary service providers substance abuse - Mental health |
|  | Other primary health care services | Does your municipality have a psychologist employed by the municipality? Yes/No |
| Involvement of volunteers in service provision |  | Does your municipality involve volunteers and/or voluntary organizations in providing long-term care services? Yes/No |
| Intermunicipal cooperation |  | Is your municipality currently involved in intermunicipal cooperation on long-term care services? Yes/No |
| Assessment of patient and family member’s satisfaction with care |  | Does your municipality conduct regular systematic patient/family surveys to assess satisfaction with the long-term care services? Yes/No |
| Use of/Purchasing services from private service providers |  | Does your municipality purchase long-term care services from private providers? Yes/No |
| Municipal long-term care plans |  | Does your municipality have a current longstanding plan for its long-term care services? Yes/No |

*Similar to the question given for a nursing home.
