# Supplementary material for: Integrating Welfare Technology in Long-term Care Services: Nationwide Cross-sectional Survey Study
Source: J Med Internet Res. 2021 Aug 16;23(8):e22316. doi: 10.2196/22316 (PMC8406104; doi:10.2196/22316)
Supplement: Multimedia Appendix 3 [file jmir_v23i8e22316_app3.doc]

# Multimedia Appendix 3 - The Phases of the thematical analysis

The qualitative data in this study were analyzed thematically according to Braun and Clarke [1] The six phases of the thematic analysis are described with examples in the table below.

| **The Phases of the thematical analysis** | **Description of the process** |
| --- | --- |
| 1. Familiarizing yourself with your data | In the first phase, the data was read and re-read, and initial ideas were noted. Here we noted preliminary themes that were found through discussing the data. These was then put aside for later. |
| 2. Generating initial codes | The second phase consisted of inductively generating initial codes systematically across the entire data.  The data relating to nursing home and home care service was kept separate. For home care service, a total of 160 codes were identified of which 71 was unique. For the nursing homes, a total of 132 codes were identified of which 58 was unique. |
| 3. Searching for themes | In the third phase, all the codes were collated into potential themes. These themes were compared with the preliminary themes conducted in phase 1. In this phase, the codes related to home care service and the nursing home were pooled together when relevant. |
| 4. Reviewing themes | We reviewed the themes with the codes and the entire data set. |
| 5. Defining and naming themes | The specifics of each theme were refined, and definitions and names for each theme was generated. The themes were:   - Types and the extent of welfare technology - From good intentions to established reality - Investing in and rigging up the welfare technology initiative - The rationale for investing in welfare technology solutions |
| 6. Producing the report | In the final phase, the report of the analysis was produced as presented in the results of the article. |

Examples from the initial thematic mapping from the analysis are presented below. An overview of included codes in different themes is available upon request.


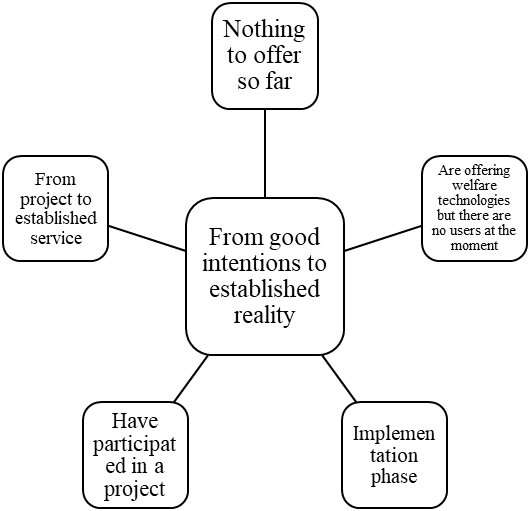


1. Braun V, Clarke V. Using thematic analysis in psychology. Qualitative research in psychology.2006;3(2):77-101.
