# Supplementary material for: Integrating Welfare Technology in Long-term Care Services: Nationwide Cross-sectional Survey Study
Source: J Med Internet Res. 2021 Aug 16;23(8):e22316. doi: 10.2196/22316 (PMC8406104; doi:10.2196/22316)

# Multimedia appendix 4 - Geels & Schot’s processual framework

Geels & Schot [1, p. 401] processual framework suggests that transitions and system changes emerge through the interactions between processes at different levels, these dynamics can be summarized as: “(a) niche-innovations gradually build up internal momentum (e.g. through learning processes, price/performance improvements, expanding social networks), (b) changes at the landscape level create pressure on the regime, (c) destabilisation of the regime creates windows of opportunity for the diffusion of niche-innovations, which aligns with ongoing regime processes leading to substantial transformation and disruption” [2, p. 1].


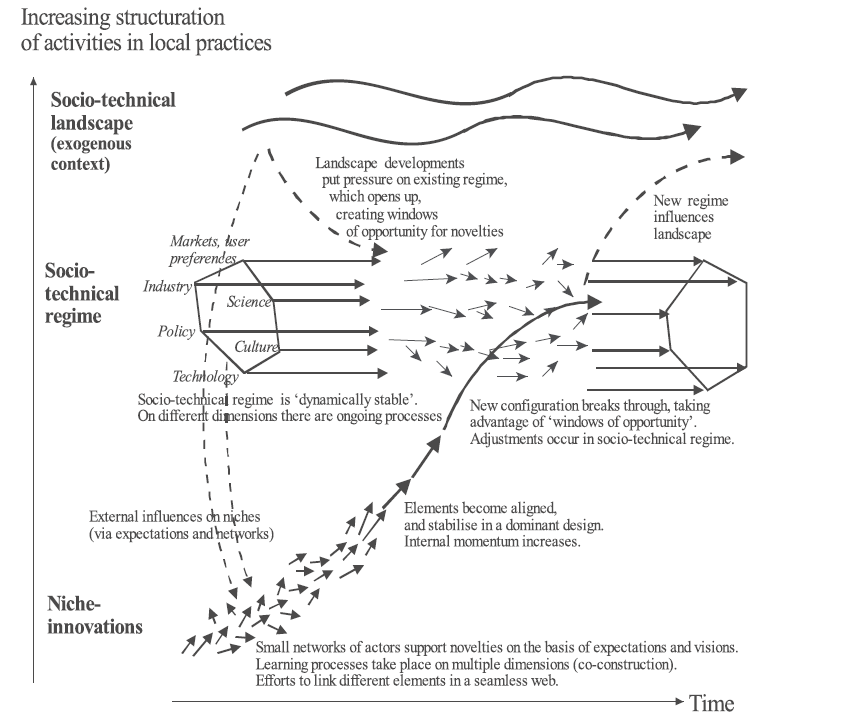

Supplement: Multimedia Appendix 4 [file jmir_v23i8e22316_app4.doc]
